# Intraoperative Use of CBCT for Identification and Localization of Calcified Canals: A Clinical Technique

**DOI:** 10.1155/2017/1265701

**Published:** 2017-11-27

**Authors:** Spyros Floratos, Maria-Elpida Miltiadous

**Affiliations:** ^1^Department of Endodontics, School of Dental Medicine, University of Pennsylvania, Philadelphia, PA, USA; ^2^Department of Endodontics, School of Dentistry, University of Athens, Athens, Greece

## Abstract

Localization of calcified canals has always been a challenge in the field of endodontics. The following report of three cases describes a technique for the identification and negotiation of obliterated canals by use of cone beam computed tomography (CBCT) intraoperatively. Canal orifices could not be found clinically in all three cases. Gutta-percha points were placed and compacted at the position where the canal orifices were estimated to be. Intraoperative CBCT was taken, and the distance between the gutta-percha points and the canal orifices was calculated at all planes of space in the first two cases. In the third case, only one canal orifice could be identified due to obliteration of the other canals.

## 1. Introduction

Cone beam computed tomography (CBCT) produces three-dimensional scans of the dentition, the maxillofacial skeleton, and the relationship of anatomic structures. Endodontic applications of CBCT include diagnosis of periapical bone defects [1], assessment of internal and external root resorption [1], detection of vertical root fractures [2], visualization and localization of perforations [3], identification of root canals, and treatment planning of surgical and complex cases such as invaginated teeth [4, 5].

The size of the field of view (FOV) can vary among different CBCT scanners from 3 to 4 cm up to 20 cm [6]. Typically the resolution of large FOV images is lower compared to that of small size FOV [7]. For endodontic purposes, the FOV should be limited to the region of interest; that is, the FOV should encompass the tooth (or teeth) under investigation and its surrounding structures [8].

The intraoperative use of CBCT for the assessment and management of calcified canals has recently been reported [9]. When canals are identified but are subjected to calcification, intraoperative CBCT has been shown to be useful for assessing the extent of calcification, thereby contributing to determining the proper sequence of the treatment [9].

Recently, intraoperative use of limited FOV for intraappointment identification and localization of calcified canals was recommended [10]; however, a clinical technique for the exact localization of the calcified canal has not been reported. In the present study, gutta-percha (GP) points are used intraoperatively as markers, and the distance between the marker and the patent canal orifice is calculated at all planes of space. A case is also presented where the same technique was performed but not all canal orifices could be identified as they were obliterated.

## 2. Cases Presentation

### 2.1. Case 1

**Figure 1:**
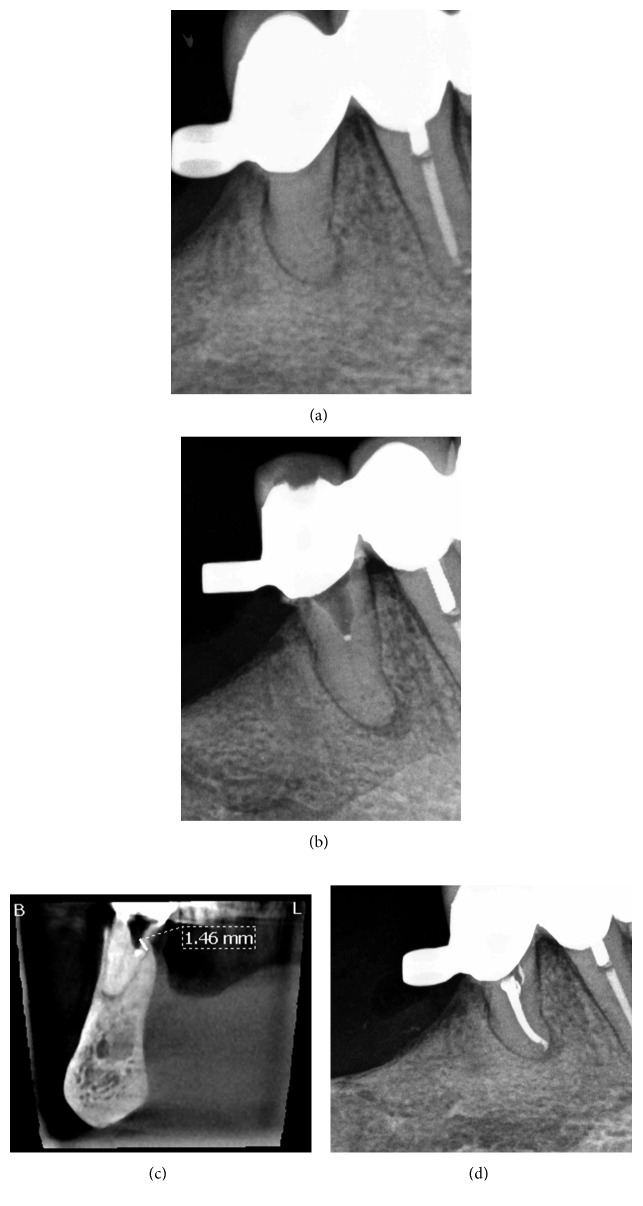
Treatment sequence of tooth 45. (a) Preoperative radiograph. (b) Intraoperative radiograph with gutta-percha point. (c) Coronal view of intraoperative CBCT with gutta-percha point. (d) Postoperative radiograph.

An 83-year-old female was referred for evaluation and treatment of her right mandibular second premolar. Clinical examination revealed pain on percussion and palpation. Periodontal probings were within normal limits, and there was no mobility. The tooth was an abutment tooth, and there was an attachment on the crown for a removable partial denture. The patient's general practitioner reported previous intraoral swelling on the buccal side of the tooth and administered antibiotics (Augmentin 625 mg three times per day for 1 week). Radiographic examination revealed canal obliteration and a periapical radiolucency of the tooth (Figure 1(a)). The patient's medical history included bisphosphonates per os (Fosamax 70 mg once per week for 10 years). A diagnosis of pulp necrosis and symptomatic apical periodontitis was established. The patient was offered all treatment options including root canal treatment with or without replacement of the prosthetic restoration or extraction of the tooth, and she decided to have root canal treatment through the crown as she did not want to have a new prosthetic restoration. The patient provided written consent for the treatment plan.

After anesthesia with 4% septocaine with 1 : 100,000 adrenaline (Ubistesin Forte, 3M ESPE, Seefeld, Germany) and rubber dam isolation (Hygenic Dental Dam, Coltene Whaledent, Langenau, Germany), an access cavity through the crown was prepared. The pulp chamber was presented with calcification, and no canal orifice could be identified under magnification. Ultrasonic preparing was performed at the depth of the preparation in an attempt to locate the canal orifice, but it was not successful. Following that, a 0.5 mm in diameter GP point was placed and compacted at the depth of the preparation where the canal orifice was estimated to be (Figure 1(b)). A cotton pellet moistened with sodium hypochlorite was placed in the access cavity, and the tooth was temporarily sealed with 4 mm of Cavit G (3M ESPE, Seefeld, Germany). The patient was referred for limited FOV CBCT of the area of the tooth (Morita 3D Accuitomo 170, J. Morita MFG Corp., Irvine, CA, USA). The coronal view of the CBCT revealed that the canal orifice was located 1.46 mm buccally and coronally to the GP point (Figure 1(c)). At the second visit, temporary filling materials were removed and ultrasonic preparing was performed buccally to the GP point at the position indicated by the CBCT. A microopener No. 10/.04 (Dentsply Maillefer, Tulsa, OK, USA) was introduced into an opening located along the buccal wall, and the canal location was confirmed using an apex locator (Root ZX II, J. Morita MFG Corp., Irvine, CA, USA). Root canal preparation was carried out using an endodontic rotary instrumentation system (RaCe, FKG Dentaire SA, La Chaux-de-Fonds, Switzerland) up to a 40/.04 file. Sodium hypochlorite 2.5% was used for copious irrigation. The canal was filled with GP and epoxy sealer (AH Plus, Dentsply Maillefer, Tulsa, OK, USA) using vertical compaction. The access cavity along with the space created lingually with ultrasonic preparing was sealed with composite resin, and a postoperative radiograph was taken (Figure 1(d)). It was not possible to recruit the patient for a yearly recall due to her advanced age.

### 2.2. Case 2

**Figure 2:**
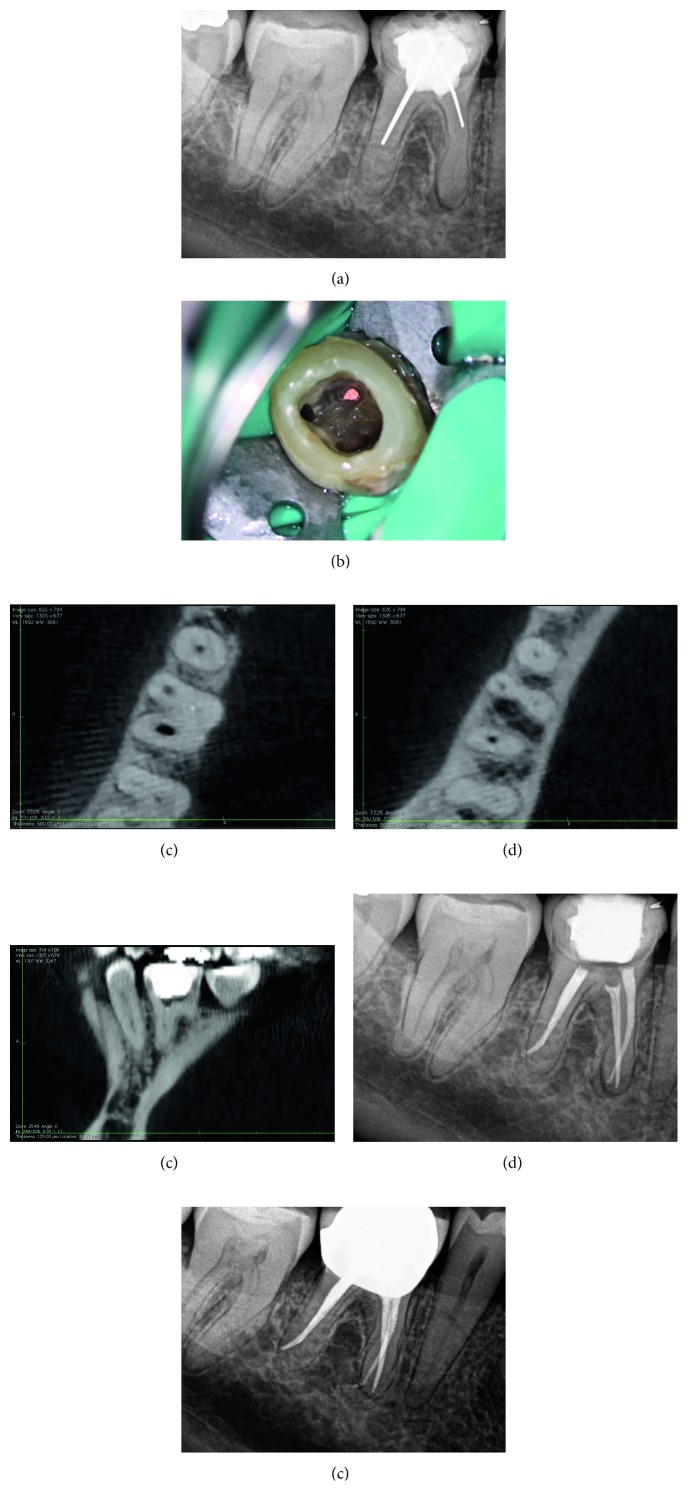
Treatment sequence of tooth 46. (a) Preoperative radiograph. (b) Clinical picture of gutta-percha point at the estimated position of the orifice of the mesiobuccal canal. (c) Axial view of intraoperative CBCT with gutta-percha point. (d) Axial view of intraoperative CBCT 2 mm apically to the gutta-percha point. (e) Sagittal view of intraoperative CBCT with gutta-percha point. (f) Postoperative radiograph. (g) Radiograph at two-year follow-up.

A 47-year-old female was referred by her general practitioner for evaluation and retreatment of her right mandibular first molar. Clinical examination revealed no symptoms. Periodontal probings were within normal limits, and there was no mobility. Radiographic examination revealed previous root canal treatment with silver points and a periapical radiolucency of the tooth. Mesial canals were obliterated and not visible (Figure 2(a)). The tooth was restored with a composite resin buildup. The patient's medical history was noncontributory. A diagnosis of previous root canal treatment with asymptomatic apical periodontitis was established. The patient was informed that conventional root canal retreatment followed by a crown was the best option. The patient consented to the treatment plan.

After anesthesia with 4% septocaine with 1 : 100,000 adrenaline (Ubistesin Forte, 3M ESPE, Seefeld, Germany) and rubber dam isolation (Hygenic Dental Dam, Coltene Whaledent, Langenau, Germany), the coronal restoration and all root canal filling materials were removed from the mesiolingual and distal canals, but the orifice of the mesiobuccal canal could not be located clinically under the dental operating microscope. Ultrasonics were used to treph along the isthmus area at a position where the orifice of the mesiobuccal canal was estimated to be. After trephing a 1 mm deep groove at the mesiobuccal canal position, the orifice could not be located. A GP point was placed and compacted at the depth of the groove (Figure 2(b)). Following that, a cotton pellet moistened with sodium hypochlorite was placed, and the access cavity was temporarily sealed with 4 mm of Cavit G (3M ESPE, Seefeld, Germany). The patient was referred for limited FOV CBCT (Morita 3D Accuitomo 170, J. Morita MFG Corp., Irvine, CA, USA). The axial view of the CBCT revealed that the canal was located 0.5 mm buccally to the GP point, and calcification was evident (Figure 2(c)). Furthermore, the canal seemed to be patent 2 mm apically to the GP point (Figure 2(d)). The sagittal view of the CBCT revealed that the orifice of the mesiobuccal canal was located 0.5 mm mesially to the GP point (Figure 2(e)). At the second visit, temporary filling materials were removed, and ultrasonic preparing was performed buccally and mesially to the GP point. A microopener No. 10/.04 (Dentsply Maillefer, Tulsa, OK, USA) was introduced into an opening located at the estimated distance according to the CBCT, and the presence of the canal was confirmed using an apex locator (Root ZX II, J. Morita MFG Corp., Irvine, CA, USA). Root canal preparation was carried out using an endodontic rotary instrumentation system (Race, FKG Dentaire SA, La Chaux-de-Fonds, Switzerland) up to a 35/0.4 file. Sodium hypochlorite 2.5% was used for copious irrigation. The canal was filled with GP and epoxy sealer (AH Plus, Dentsply Maillefer, Tulsa, OK, USA) using warm vertical compaction. The access cavity was sealed with composite resin, and a postoperative radiograph was taken (Figure 2(f)). The patient was recalled yearly for clinical and radiographic follow-ups. At two-year follow-up, the tooth was asymptomatic, and periapical healing was observed radiographically (Figure 2(g)).

### 2.3. Case 3

**Figure 3:**
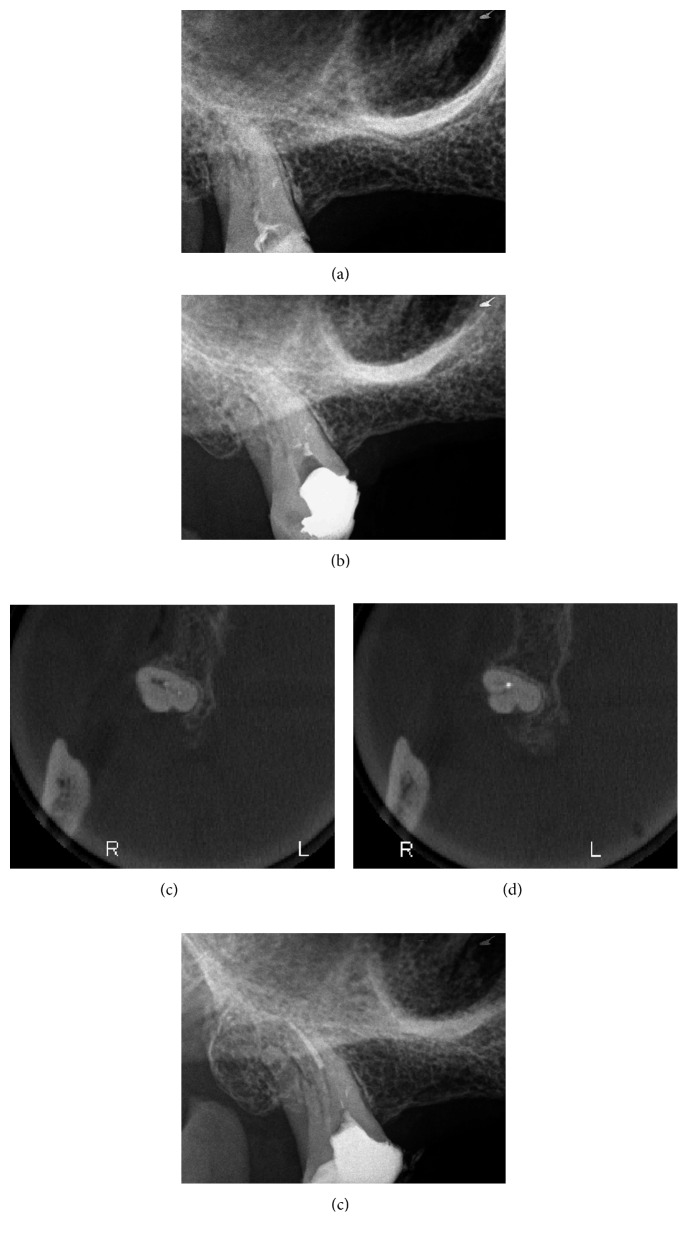
Treatment sequence of tooth 18. (a) Preoperative radiograph. (b) Intraoperative radiograph with gutta-percha points. (c) Axial view of intraoperative CBCT with both gutta-percha points. (d) Axial view of intraoperative CBCT with mesiobuccal gutta-percha point. (e) Postoperative radiograph.

A 55-year-old male was referred for evaluation and treatment of his right maxillary third molar. The referring prosthodontist reported that the tooth had symptoms on biting and made an access cavity and placed a temporary restoration as an emergency treatment. The tooth would be used as an abutment tooth for a fixed partial denture. Clinical examination revealed a temporary restoration on the tooth and no symptoms upon percussion or palpation. Periodontal probings were within normal limits, and there was no mobility. Radiographic examination revealed canal obliteration and no apparent periapical radiolucency of the tooth (Figure 3(a)). The patient's medical history was noncontributory. A diagnosis of asymptomatic irreversible pulpitis was established. A written consent was given by the patient for endodontic treatment of the tooth.

After anesthesia with 4% septocaine with 1 : 100,000 adrenaline (Ubistesin Forte, 3M ESPE, Seefeld, Germany) and rubber dam isolation (Hygenic Dental Dam, Coltene Whaledent, Langenau, Germany), the coronal restoration was removed, and an access cavity was prepared. The pulp chamber was presented with calcification, and no canal orifices could be identified clinically under the dental operating microscope. Ultrasonic preparing was performed in an attempt to locate the canal orifices, but it was not successful. Following that, two GP points were placed and compacted where the orifices of the mesiobuccal and palatal canals were estimated to be (Figure 3(b)). A cotton pellet moistened with sodium hypochlorite was placed in the access cavity, and the tooth was temporarily sealed with 4 mm of Cavit G (3M ESPE, Seefeld, Germany). The patient was referred for limited FOV CBCT of the tooth (Morita 3D Accuitomo 170, J. Morita MFG Corp., Irvine, CA, USA). The axial view of the CBCT revealed that the palatal canal orifice was located at the GP placed on the palatal position whereas the mesiobuccal canal was located buccally to where the respective GP point was placed (Figures 3(c) and 3(d)). At the second visit, temporary filling materials were removed, and ultrasonic preparing was performed according to the information from the CBCT. By use of a microopener No. 10/.04 (Dentsply Maillefer, Tulsa, OK, USA), the palatal canal orifice was found and confirmed using an apex locator (Root ZX II, J. Morita MFG Corp., Irvine, CA, USA). The orifices of the mesiobuccal and distobuccal canals could not be located. Root canal preparation of the palatal canal was carried out using an endodontic rotary instrumentation system (Race, FKG Dentaire SA, La Chaux-de-Fonds, Switzerland) up to a 40/0.4 file. Sodium hypochlorite 2.5% was used for copious irrigation. The canal was filled with GP and epoxy sealer (AH Plus, Dentsply Maillefer, Tulsa, OK, USA) using warm vertical compaction. Preparation of a post was made on the palatal canal. The access cavity was temporarily sealed with cotton pellet and Cavit G, and a postoperative radiograph was taken (Figure 3(e)). The tooth was followed up for a month and remained asymptomatic. After that, the patient was referred back to the prosthodontist.

## 3. Discussion

Calcific metamorphosis or calcification is a pulpal response to trauma characterized by rapid deposition of hard tissue within the canal space [11]. In most cases, calcification appears to be severe coronally, tapering off towards the root apex [12]. Teeth with severe pulp calcification and root canal obliteration can complicate the access cavity preparation and root canal identification and negotiation, thus leading to unnecessary dentin removal and iatrogenic mishaps such as perforations. In this study, ultrasonic preparing of the dentinal structure was performed, and GP points were placed at the point where the canal orifice was estimated to be. Following that, a limited FOV CBCT was taken, and the GP point appeared as a radiopaque marker on the CBCT. Resilon points (RealSeal, SybronEndo, Amersfoort, Netherlands) could also be used as they are more radiopaque than GP. In order for the GP point to be visible, it should be at least 0.5 mm in diameter. If a permanent coronal restoration is present on the tooth (e.g., amalgam and crown), then the GP point should be placed 2 mm apically to the CEJ; otherwise, its position will be difficult to visualize because of the scattering caused by the coronal restoration. The technique described allows for a measurement of the distance between the GP marker and the point where the canal is visible. This leads to a more accurate estimation of the canal opening in the clinical situation. As the resolution of the computed tomography visualization is enhanced, a limited FOV scan will probably reveal different levels of calcification all along the canal in the near future. Based on the radiopacity of different levels of calcification, an estimation of the point whether the canal is patent will probably be possible.

Different ways to locate canal orifices have been reported in the literature. The use of illumination and magnification provided by the dental operating microscope leads to easier and safer identification of canal orifices [13]. Knowledge and understanding of the anatomy and color differences of the canal chamber floor can assist the clinician in the identification of canal orifices [14]. Other aids to locate root canals include staining the chamber floor with 1% methylene blue dye, additional preoperative radiographs from different directions, looking for canal bleeding points, and performing the sodium hypochlorite “champagne bubble” test [15].

The intraoperative use of CBCT in complicated endodontic cases can assist the clinician in ensuring a safer procedure and a more predictable treatment outcome. However, the use of ionizing radiation should always comply with the principle of ALARA (As Low As Reasonably Achievable) in order to minimize the patients' exposure to radiation [16]. Therefore, clinicians must take into consideration the effective dose and imaging quality of various imaging techniques and optimize all the diagnostic information provided by lower radiation conventional radiographs before turning to CBCT.

In the cases reported in our study, conventional periapical radiographs and the dental operating microscope could not provide sufficient information on the location of calcified canal orifices. Therefore, the intraoperative use of CBCT was necessary and proved to be vital in successfully locating the calcified canal orifices. However, a maxillary third molar case is also reported where the buccal canals were completely calcified and could not be located even with the intraoperative use of CBCT. A limited FOV was used, thus reducing the effective dose to the patient and producing higher resolution images. Additional research on the intraoperative applications of CBCT in endodontics and their impact on the treatment outcome will be needed.
